# Detection of Mtb and NTM: preclinical validation of a new asymmetric PCR-binary deoxyribozyme sensor assay

**DOI:** 10.1128/spectrum.03506-23

**Published:** 2024-04-23

**Authors:** Yasmin Castillos das Neves, Ana Julia Reis, Marcos Alaniz Rodrigues, Erica Chimara, Maria Cristina da Silva Lourenço, Jacques Fountain, Ivy Bastos Ramis, Andrea von Groll, Yulia Gerasimova, Kyle H. Rohde, Pedro Eduardo Almeida da Silva

**Affiliations:** 1 Laboratory of Mycobacteria, Núcleo de Pesquisa em Microbiologia Médica, Universidade Federal do Rio Grande, Rio Grande do Sul, Brazil; 2 Rede Brasileira de Pesquisa em Tuberculose (REDE-TB), Rio Grande, Rio Grande do Sul, Brazil; 3 Instituto Adolfo Lutz, São Paulo, Brazil; 4 Fundação Oswaldo Cruz (Fiocruz), Rio de Janeiro, Brazil; 5 Division of Immunity and Pathogenesis, Burnett School of Biomedical Sciences, College of Medicine, University of Central Florida, Orlando, Florida, Orlando, USA; 6 Department of Chemistry, College of Sciences, University of Central Florida, Orlando, Florida, Orlando, USA; Johns Hopkins University School of Medicine, Baltimore, Maryland, USA

**Keywords:** diagnostic method, deoxyribozymes, *Mycobacterium*, *M. tuberculosis*, non-tuberculous mycobacteria

## Abstract

Tuberculosis (TB) and infectious diseases caused by non-tuberculous mycobacteria (NTM) are global concerns. The development of a rapid and accurate diagnostic method, capable of detecting and identifying different mycobacteria species, is crucial. We propose a molecular approach, the BiDz-TB/NTM, based on the use of binary deoxyribozyme (BiDz) sensors for the detection of *Mycobacterium tuberculosis* (Mtb) and NTM of clinical interest. A panel of DNA samples was used to evaluate Mtb-BiDz, *Mycobacterium abscessus*/*Mycobacterium chelonae*-BiDz, *Mycobacterium avium*-BiDz, *Mycobacterium intracellulare*/*Mycobacterium chimaera*-BiDz, and *Mycobacterium kansasii*-BiDz sensors in terms of specificity, sensitivity, accuracy, and limit of detection. The BiDz sensors were designed to hybridize specifically with the genetic signatures of the target species. To obtain the BiDz sensor targets, amplification of a fragment containing the hypervariable region 2 of the 16S rRNA was performed, under asymmetric PCR conditions using the reverse primer designed based on linear-after-the-exponential principles. The BiDz-TB/NTM was able to correctly identify 99.6% of the samples, with 100% sensitivity and 0.99 accuracy. The individual values of specificity, sensitivity, and accuracy, obtained for each BiDz sensor, satisfied the recommendations for new diagnostic methods, with sensitivity of 100%, specificity and accuracy ranging from 98% to 100% and from 0.98 to 1.0, respectively. The limit of detection of BiDz sensors ranged from 12 genome copies (Mtb-BiDz) to 2,110 genome copies (Mkan-BiDz). The BiDz-TB/NTM platform would be able to generate results rapidly, allowing the implementation of the appropriate therapeutic regimen and, consequently, the reduction of morbidity and mortality of patients.

**IMPORTANCE:**

This article describes the development and evaluation of a new molecular platform for accurate, sensitive, and specific detection and identification of *Mycobacterium tuberculosis* and other mycobacteria of clinical importance. Based on BiDz sensor technology, this assay prototype is amenable to implementation at the point of care. Our data demonstrate the feasibility of combining the species specificity of BiDz sensors with the sensitivity afforded by asymmetric PCR amplification of target sequences. Preclinical validation of this assay on a large panel of clinical samples supports the further development of this diagnostic tool for the molecular detection of pathogenic mycobacteria.

## INTRODUCTION

Tuberculosis (TB), caused mainly by *Mycobacterium tuberculosis*, remains a disease of healthcare concern with an estimated 10.6 million new cases and 1.3 million deaths in 2022. According to the World Health Organization (WHO), per year, about four million people with TB are not diagnosed and/or treated, representing a major obstacle for TB control due to the *M. tuberculosis* transmission maintenance (1).

Besides the impact of TB on global health, an increasing incidence of pulmonary diseases caused by non-tuberculous mycobacteria (NTM) has been observed, which, although being ubiquitous and environmental microorganisms, are considered opportunistic pathogens (2, 3). In general, 90% of infections caused by NTM are pulmonary, which may present clinical manifestations like TB, showing high morbidity and mortality. Members of the *Mycobacterium avium* complex (MAC), *Mycobacterium abscessus* complex (MABC), and *Mycobacterium kansasii* are the most frequently isolated NTM in pulmonary infections in the world (4–6).

The main clinically important MAC species are *M. avium*, *Mycobacterium intracellulare*, and *Mycobacterium chimaera*. Although infectious diseases caused by all MAC species have the same treatment, differentiation of the members of this complex is epidemiologically important. This is because *M. avium* is responsible for most cases of infectious diseases caused by NTM among HIV-positive patients, who are less responsive to treatment, while *M. intracellulare* and *M. chimaera* are more prevalent among immunocompetent patients (7–11).

MABC, besides being considered the major cause of pulmonary infections among the fast-growing mycobacteria, is associated with high rates of antimicrobial resistance, which makes the treatment of infectious diseases caused by this complex a clinical challenge (9, 12–14). Another pathogenic fast-growing mycobacteria, with high genomic similarity to MABC, is *Mycobacterium chelonae*. The genetic similarity between MABC members and *M. chelonae* resulted in the grouping of these species until 1992 (15, 16), when members of the MABC were considered an isolated species, forming the *M. chelonae*-*abscessus* complex (17). Finally, the separation of these species and MABC creation was proposed in 2009 (18). Despite their close relation and the possibility of using the same therapeutic scheme to treat infectious diseases caused by these mycobacteria, *M. chelonae* is a less common cause of pulmonary infections, when compared with MABC (19, 20).

*M. kansasii* is among the most pathogenic and clinically relevant NTMs, capable of causing aggressive TB-like pulmonary infections. Compared to the pulmonary infections caused by the NTM described above, *M. kansasii* pulmonary infections are easily treated and generally responsive to anti-TB treatment (7, 21–23). The overlap in clinical features observed between TB and infections caused by *M. kansasii* can be explained by the phylogenetically close relationship of the latter to the common ancestor of the *Mycobacterium tuberculosis* complex (MTBC), with which it shares numerous genes, including virulence factors (23–25).

In routine medical practice, TB diagnosis is based on clinical criteria, chest radiological findings, and microbiological approach (26). However, the diagnosis of pulmonary diseases caused by NTM is challenging due to the difficulties in distinguishing, both clinically and radiologically, infectious diseases caused by NTM from TB (7, 27, 28). Moreover, the current diagnostic methods have some limitations, such as low sensitivity and specificity (especially for smear microscopy), high cost per test, dependence on the limited number of suppliers, need for complex laboratory infrastructure, cumbersome methods, and a long turnaround time (29–34).

The abovementioned concerns highlight the need to develop diagnostic methods that have a point-of-care (POC) profile, such as high specificity and sensitivity, fast and easy execution, and low cost (35). Herein, we propose a molecular approach based on a novel platform combining linear-after-the-exponential (LATE)-PCR, an asymmetric PCR method for optimal generation of ssDNA analytes (36, 37), and binary deoxyribozyme (BiDz) sensors to identify the *M. tuberculosis* complex and the main clinically important NTM species.

Previous studies evaluating the BiDz sensors have shown that this method offers advantages that make it a promising point-of-care platform, such as high sensitivity, low-cost, and easy-to-synthesize reagents (38–40). Thereby, the objective of this study was to evaluate the specificity, sensitivity, accuracy, and DNA detection limit of the BiDz-TB/NTM methodology in the detection and identification of *M. tuberculosis* and the NTM species *M. abscessus* complex/*M. chelonae*, *M. avium*, *M. intracellulare*/*M. chimaera*, and *M. kansasii*.

## MATERIALS AND METHODS

### Study design

To evaluate the BiDz-TB/NTM method, 41 mycobacterial strains were included in this study. These strains were obtained from the strain biobank of the Laboratory of Mycobacteria, Núcleo de Pesquisa em Microbiologia Médica of the Universidade Federal do Rio Grande (Rio Grande, Rio Grande do Sul), of the Instituto Adolfo Lutz (São Paulo, São Paulo) and of the Laboratório de Pesquisa em Resistência Bacteriana of the Centro de Pesquisas Experimentais (Porto Alegre, Rio Grande do Sul).

The encompassed strains were *M. tuberculosis* complex (6 of 41), *M. abscessus* subsp. *abscessus* (5 of 41), *M. abscessus* subsp. *bolletii* (5 of 41), *M. abscessus* subsp. *massiliense* (5 of 41), *M. avium* (6 of 41), *M. intracellulare* (4 of 41), *M. kansasii* (5 of 41), *M. fortuitum* (1 of 41), *M. marinum* [(*Mycobacterium marinum* Aronson - ATCC 927), *M. chelonae* (ATCC 946), *M. chimaera* (1 of 41), and *Mycobacterium szulgai* (ATCC 10831). In addition, six strains of Gram-positive and Gram-negative bacteria were included: *Vibrio coralliilyticus* (clinical strain), *Escherichia coli* (ATCC 25922), *Staphylococcus aureus* (ATCC 12598), *Acinetobacter baumannii* (ATCC 19606), *Pseudomonas aeruginosa* (ATCC 15442), and *Salmonella enterica* serovar Typhimurium (ATCC 14028). All assays were performed in duplicate.

Except for the ATCC strains, all strains were previously identified using the *rpo*B and *hsp*65 genes sequencing, for NTM strains, and mycobacterial interspersed repetitive unit-variable number tandem repeat 24-loci, insertion sequence (IS)*6110* and whole-genome sequencing for *M. tuberculosis* strains (Table S1, supplemental material).

### Genomic DNA extraction

Strains stored at −20°C were cultured in solid Ogawa-Kudoh media (*Mycobacterium* sp.) or nutrient agar (Gram-positive and Gram-negative bacteria) and incubated at 37°C until bacterial growth. Bacterial colonies (two to three loops of 0.5-cm diameter) were added to a tube containing 500 µL of 1× Tris-EDTA buffer and subjected to thermal inactivation at 85°C for 30 minutes. All inactivated samples were submitted to the cetyltrimethylammonium bromide/NaCl method (41) and stored at −20°C until its use.

### BiDz-TB/NTM method principle

**Fig 1:**
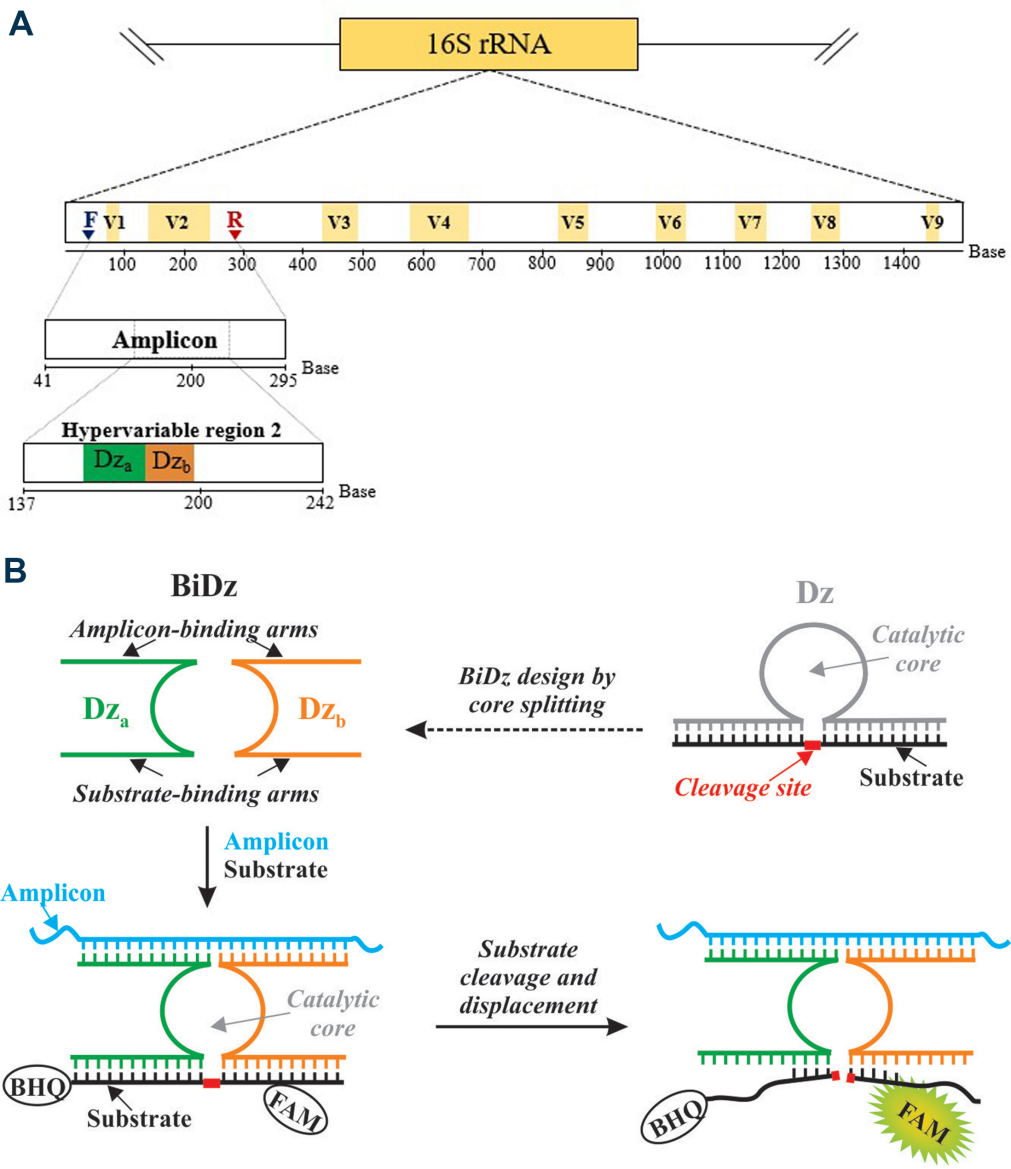
Principle of binary deoxyribozyme (BiDz) sensors. (**A**) Representation of the amplified DNA analyte indicating the binding sites for forward (blue) and reverse (red) primers in the conserved regions of the *rrs* gene encoding for 16S rRNA, as well as the sites of the hypervariable region 2 (V2) of 16S rRNA interrogated by the BiDz sensors (Dz_a_, green; Dz_b_, orange). The numbering of hypervariable regions is based on the *Escherichia coli* nomenclature system (42). (**B**) Binding of BiDz sensors to the target DNA and of the fluorogenic substrate (MzF-FAM) to the BiDz sensors, resulting in the catalytic core formation, MzF-FAM cleavage, and fluorescence emission.

The BiDz-TB/NTM is based on the use of BiDz sensors composed of two subunits (Dz_a_ and Dz_b_) with the target-binding fragments complementary to the second hypervariable region (V2) of the *rrs* gene encoding 16S rRNA from MTBC (Mtb-BiDz sensor), MABC, and *M. chelonae* [*M*. *abscessus* (Mab)/Mche-BiDz sensor], *M*. *avium* (Mav-BiDz sensor), *M. intracellulare* and *M. chimaera* (Mint/Mchi-BiDz sensor), and *M. kansasii* (Mkan-BiDz sensor) (Fig. 1A) (38). The BiDz sensors, in the presence of specific target DNA, form a catalytic core that can cleave a phosphodiester bond between two ribonucleotides present in the fluorogenic substrate (MzF-FAM). The substrate is equipped with a fluorescein (FAM) fluorophore and a Black Hole quencher at the opposite sides from the cleavage site, so that the target-induced cleavage results in a fluorescent signal (Fig. 1B; Table S2, supplemental material).

### Experimental activities

#### Asymmetric PCR

##### Optimization of LATE-PCR amplification of mycobacterial 16S rRNA

To allow for identification of clinically important mycobacterial species, a previously validated hypervariable region of the 16S rRNA gene containing species-specific sequence motifs was exploited as the target analyte for BiDz sensors (38). A pair of universal primers for species of the genus *Mycobacterium* sp. was designed to amplify the ~250-bp variable region (Fig. 1S, supplemental material). The forward primer is complementary to the conserved fragment that precedes V1 of the 16S rRNA, and reverse primers are complementary to the conserved fragment between V2 and V3 of the 16S rRNA (Fig. 1).

To validate the utility of applying LATE-PCR primer design principles (36, 37), which compensate for the reduced effective annealing of the limiting primer, three different reverse primers were evaluated (Table 1). These included the primer R2 (designed with *T*_*m*_ ~forward primer) for symmetric PCR, R + 3 (LATE primer with *T*_*m*_ = 3° >R2) and R + 5 (LATE primer with *T*_*m*_ = 5° >R2). PCRs under symmetric (forward and R2 reverse primers at 1-µM concentrations) and asymmetric [reverse primer at 1:40 dilution (0.025 µM concentration)] conditions were evaluated using *M. tuberculosis* chromosomal DNA as the template. The selective production of single-stranded DNA amplicons after the limiting primer is depleted results in analytes that are ideal for hybridization-based detection with BiDz sensors.

**TABLE 1:** Set of primers used for 16S rRNA fragment amplification

Primer	Sequence	Reference
Forward	5′-GGCGTGCTTAACACATGCA-3′	(38)
R2 reverse	5′-CCGGCTACCCGTCGTC-3′	(38)
R + 3 reverse	5′-**GG**CCGGCTACCCGTCGTC-3′	This study
R + 5 reverse	5′-**CTCAGG**CCGGCTACCCGTCGTC-3′	This study

To determine the effect of different reverse primer lengths on amplification efficiency (based on BiDz signal levels), threefold serial dilutions of DNA (10.0–0.12 ng) were tested. Additional optimization of the LATE-PCR assay (annealing temperature, type of DNA polymerase, PCR cycle number, and primer concentration for symmetric and asymmetric conditions) was also conducted (data not shown). After optimizations, R2 and LATE R + 5 primers were evaluated under asymmetric conditions using chromosomal DNA from *M. abscessus* subsp. *abscessus*, *M. abscessus* subsp. *bolletii*, *M. abscessus* subsp. *massiliense*, *M. avium*, *M. intracellulare*, and *M. kansasii* as template. Based on data showing optimal performance of LATE R + 5 primer, all subsequent tests were performed using asymmetric LATE PCR (LATE-PCR) as detailed below.

PCR reactions were performed using 10 µL of Phusion High-Fidelity PCR Master Mix kit (Thermo Scientific), 2 µL of forward primer (final concentration: 1 µM), 0.5 µL of LATE R + 5 primer (final concentration: 25 nM), and 1 µL of DNA analyte (0.75 ng DNA, final concentration: 37.5 pg/µL) to a final volume of 20 µL. All DNAs were quantified using the BioDrop spectrophotometer. The following PCR conditions were used: 98°C for 20 seconds, 45 cycles of 98°C for 5 seconds, 65°C for 10 seconds, 72°C for 5 seconds, and 72°C for 1 minute.

### BiDz-TB/NTM method using BiDz sensors

After DNA amplification, PCR products were added to 96-well plates containing the species-specific BiDz sensors and the fluorescent substrate MzF-FAM (FAM fluorophore: Abs λmax = 495 nm; Em. λmax = 520 nm). The plates were incubated at 55°C for 30 minutes in the StepOnePlus Real-Time PCR equipment, and the emitted fluorescence was subsequently read. Assays were performed using 15 µL of pH 8.0 buffer (50 mmol/L of HEPES, pH 8.0, 50 mmol/L of MgCl_2_, and 1% dimethyl sulfoxide), 0.45 µL of Dz_a_ and Dz_b_ subunits of each BiDz sensor set (1 µM), 0.6 µL of the MzF-FAM fluorescent substrate (10 µM), and 3 µL of the PCR products for a final volume of 30 µl.

#### Standardization of BiDz sensors

To increase the specificity of the BiDz sensors in the detection of *M. tuberculosis* complex members and NTM, the use of sensors in different concentrations was evaluated: 15, 20, and 25 nM. One DNA sample (15 ng/µL) was used for PCR and follow-up assay with each BiDz sensor: *M. tuberculosis* (Mtb-BiDz), *M. abscessus* subsp. *bolletii* (Mab/Mche-BiDz), *M. avium* (Mav-BiDz), *M. intracellulare* (Mint/Mchi-BiDz), and Mkan (Mkan-BiDz). Furthermore, considering the need to develop a fast, simple, and low-cost method, a reduction of the buffer constituents was also performed by removing the NaCl and KCl salts. The MgCl_2_ was maintained because it is critical for the catalytic activity of the BiDz sensors (data not shown).

### Data analysis

Absolute fluorescence values were tabulated and analyzed in Microsoft Excel. The results are presented using the signal:background ratio (S:B). Samples with an S:B greater than 2 were considered positive. The background (B) was defined as the absolute fluorescent signal (S) obtained only in the presence of the BiDz sensors and the fluorescent substrate (38).

### Specificity, sensitivity, and accuracy evaluation

To evaluate the specificity and sensitivity of the BiDz sensors individually and of the BiDz-TB/NTM method in general, the fluorescence values were tabulated, and the fluorescence values obtained for targeted and non-targeted bacterial species were compared. Samples with S:B >2 were considered positive.

The specificity of the BiDz sensors was determined by calculating 100% × *d* / (*b* + *d*). To determine the sensitivity of the sensors, the following calculation was used: 100% × *a* / (*a* + *c*). Finally, for the accuracy evaluation, the following calculation was used: (*a* + *d*) / (*a* + *b* + *c* + *d*) [*a*: S:B >2 in the presence of the target DNA analyte (true positive); *b*: S:B **>**2 in the presence of the non-target DNA analyte (false positive); *c*: S:B **<**2 in the presence of target DNA analyte (false negative); *d*: S:B <2 in the presence of non-target DNA analyte (true negative)].

### Detection limit evaluation

Genomic DNAs of *M. tuberculosis*, *M. abscessus* subsp. *abscessus*, *M. avium*, *M. intracellulare*, and *M. kansasii* were used. For each species, 20 serial twofold dilutions were tested, with an initial concentration of 15 ng/µL and a 20th concentration of 0.0286 pg/µL (quantitation performed on a Quantus portable fluorometer, using the QuantiFluor ONE dsDNA System reagent; Promega). Samples with S:B >2 were considered positive.

To determine the final limit of detection (LoD), the last concentration detected in the BiDz assays and the number of base pairs of the targeted bacterial species genomes were used [*M. tuberculosis* H37Rv: 4,411,532-bp linear DNA (GenBank: AL123456.3); *M. abscessus* ATCC 19977: 5,067,172-bp circular DNA (GenBank: CU458896.1); *M. avium*: 4,961,843-bp circular DNA (GenBank: NZ_CP028731.1); *M. intracellulare* ATCC 13950: 5,402,409-bp circular DNA (GenBank: NZ_CP076382.1); *M. kansasii* ATCC 12478: 6,432,277-bp circular DNA (GenBank: CP006835.1)].

The following calculation was used: number of copies = (ng × 6.022 × 10^23^) / (bp × 1 × 10^9^ × 650). This calculation assumes that the average weight of a base pair is 650 daltons.

## RESULTS

**Fig 2:**
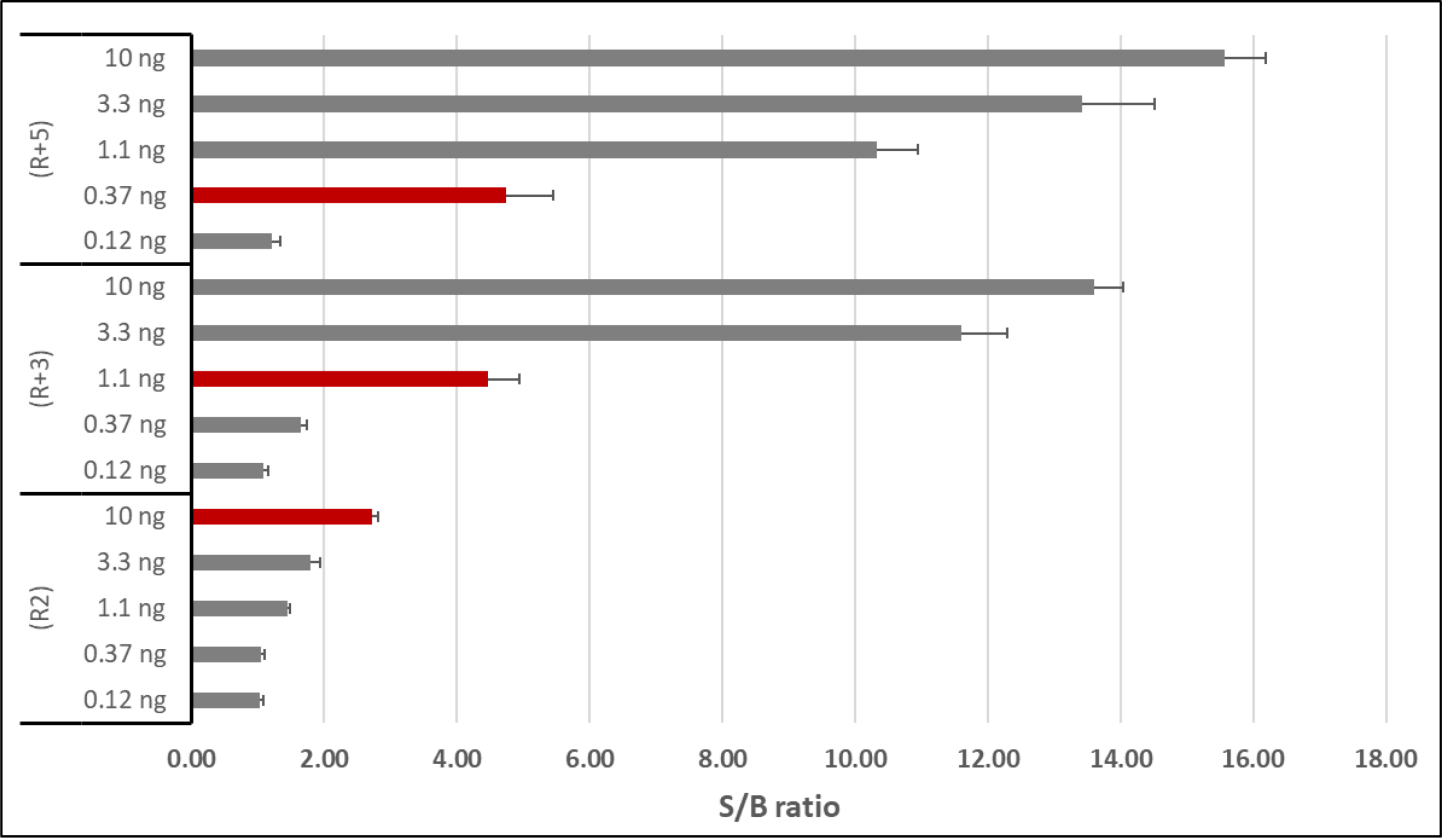
Validation and optimization of LATE asymmetric PCR primer designs. Based on LATE-PCR principles, extended-length variants of the conventional R2 reverse primer with increased annealing temperatures of 3°C (R + 3) and 5°C (R + 5) were tested. The limiting reverse primer (0.025 µM) was used at a ratio of 1:40 relative to the forward primer concentration (1 µM). Threefold serial dilutions of input template (Mtb chromosomal DNA) starting at 10ng/reaction served as templates for PCR amplification, followed by detection of BiDz sensor fluorescent signal. Signal:background ratios (S:Bs) were determined by dividing the raw fluorescent signal from the analyte-containing sample by the background signal from the sample from which template DNA was omitted. Red bars indicate the lowest input (analyte) that yielded S:B of >2.

The critical first step in our BiDz-TB/NTM diagnostic platform involves amplification of a species-specific target sequence within the 16S rRNA gene for detection by BiDz sensors. Optimal assay sensitivity requires efficient target sequence amplification and generation of detectable analytes. Consistent with previous studies, traditional asymmetric PCR using conventionally designed primers at unequal concentrations (for R2) proved to be inefficient. Using *M. tuberculosis* chromosomal DNA and Mtb-BiDz sensors, a significant fluorescent signal (signal:background >2) was only detected when 10 ng of DNA was used as template (Fig. 2). This suggests poor yield of single-stranded analyte capable of hybridization with BiDz sensor strands. Extending the length and increasing the predicted annealing temperature of the reverse primer (R + 3, R + 5) improved the sensitivity of the assay considerably, affording detection of >25-fold less DNA simply by adding 6 bp to the reverse primer (R + 5) (Fig. 2).

**Fig 3:**
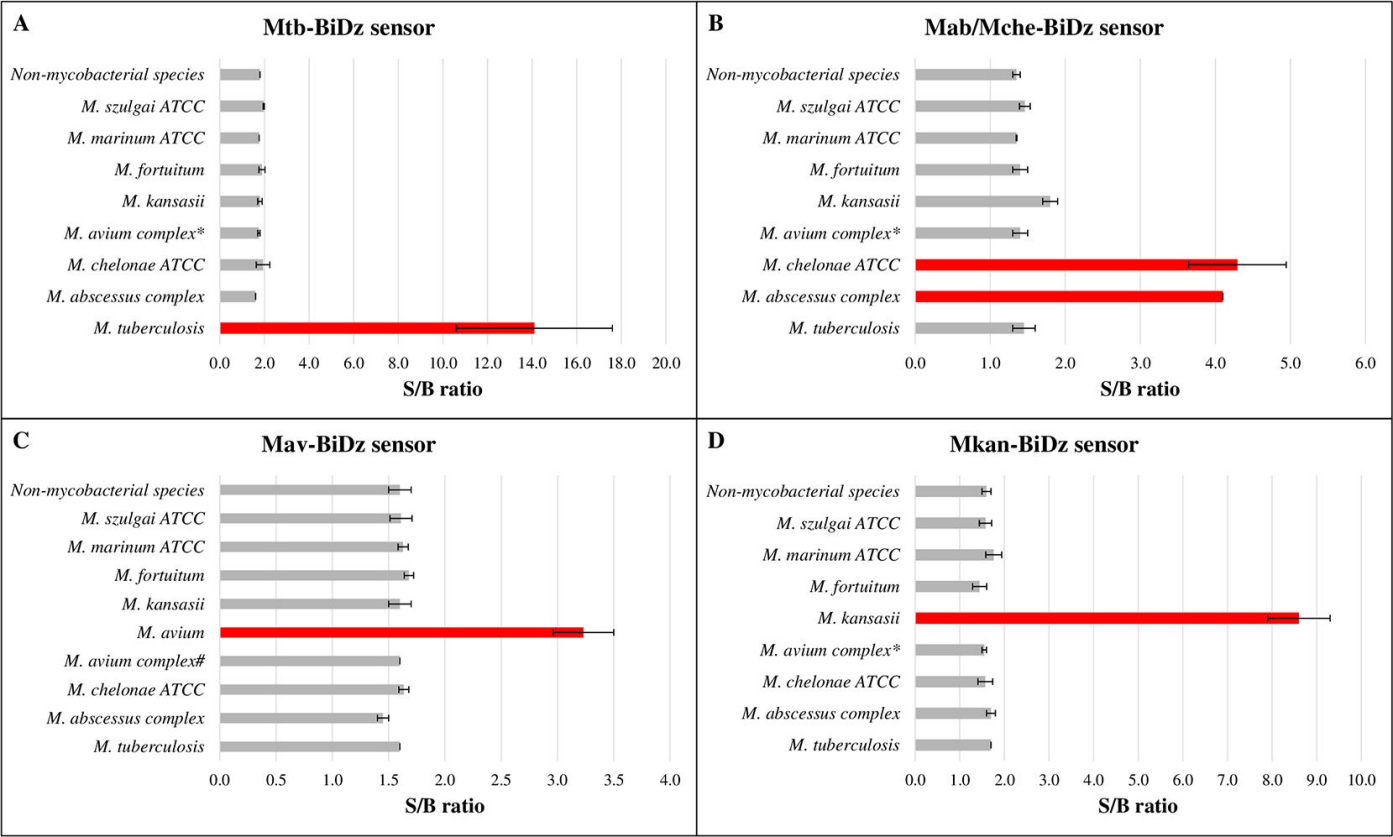
Evaluation of the specificity, sensitivity, and accuracy of BiDz sensors against target and non-target DNAs. The results of the signal:background ratio (S:B) are expressed as an absolute mean for target and non-target species. Samples with S:B of >2 were considered positive. Red bars indicate the target species of the Mtb-BiDz (**A**), Mab/Mche-BiDz (**B**), Mav-BiDz (**C**), and Mkan-BiDz (**D**) sensors. Gray bars indicate the non-target species. The results shown represent the average of all clinical isolates for each species. Note: *M abscessus complex* includes subsp. *massiliense*, *bolletii*, and *abscessus* strains. *M. avium complex species evaluated: *M. avium*, *M. intracellulare*, and *M. chimaera*. **#**Species of the *M. avium* complex evaluated: *M. intracellulare* and *M. chimaera*. **Non-mycobacterial species**: *Vibrio coralliilyticus*, *Escherichia coli*, *Staphylococcus aureus*, *Acinetobacter baumannii*, *Pseudomonas aeruginosa* and *Salmonella enterica* serovar Typhimurium. Graphs of data for individual isolates can be found in Fig. S2.

**Fig 4:**
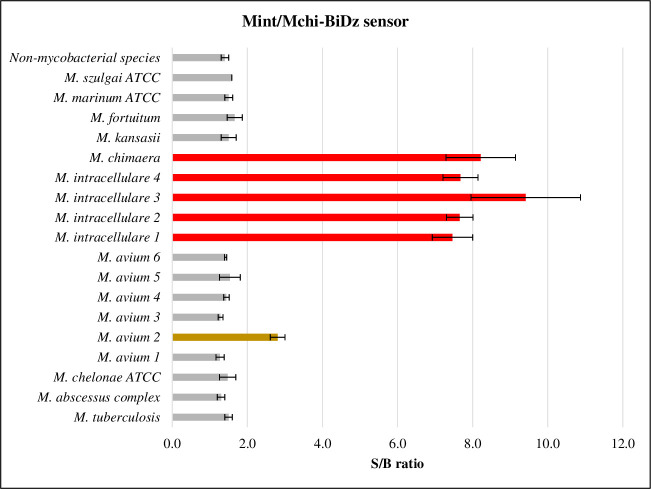
Evaluation of the specificity, sensitivity, and accuracy of the Mint/Mchi-BiDz sensor against target and non-target DNAs. The results of the signal:background ratio (S:B) are expressed as an absolute mean, except for samples of *M. intracellulare* and *M. avium*. Samples with S:B of >2 were considered positive. The red bars indicate the target species of the Mint/Mchi-BiDz sensor. The gray bars indicate the non-target species, and the *M. avium* sample with a positive result is indicated in gold. Non-mycobacterial species: *Vibrio coralliilyticus*, *Escherichia coli*, *Staphylococcus aureus*, *Acinetobacter baumannii*, *Pseudomonas aeruginosa*, and *Salmonella enterica* serovar Typhimurium.

When sensors from other species were also evaluated, a higher fluorescent signal in the BiDz assays using the PCR amplicons obtained with LATE (R + 5) versus R2 reverse primer was observed for all BiDz sensors. When tested against non-mycobacterial species, including both Gram-negative and Gram-positive pathogens, sensors for all targeted mycobacterial species yielded an S:B of ≤2, affirming the specificity of the assay (Fig. 3; Fig. 4; Fig. S2). For the targeted bacterial species, only when the LATE primer was used, positive results were obtained, with an S:B of >2 (Mab/Mche-BiDz: S:B = 2.6, Mav-BiDz: S:B = 2.1, and Mint/Mchi-BiDz: S:B = 2.5). For the Mkan-BiDz sensor, an S:B of >2 was observed regardless of the reverse prime used: S:B = 10 or S:B = 2.9 when (R + 5) or R2 was used, respectively.

Next, we calculated specificity and sensitivity of the assay as the rates of true negatives and true positives, respectively. In addition, accuracy was calculated as a fraction of correctly identified (true positive and true negative) samples. When the BiDz sensors were evaluated against LATE-PCR (R + 5) amplicons from targeted and non-targeted bacterial species, we obtained specificity, sensitivity, and accuracy of 100%, 100%, and 1.0 for the Mtb-BiDz (Fig. 3A), Mab/Mche-BiDz (Fig. 3B), Mav-BiDz (Fig. 3C), and Mkan-BiDz (Fig. 3D), and 98%, 100%, and 0.98 for Mint/Mchi-BiDz, respectively (Table 2; Fig. S2, supplemental material). For Mint/Mchi-BiDz, an intermediate fluorescence signal was observed for a *M. avium* DNA sample (*M. avium* 2: S:B = 2.8: Fig. 4), which resulted in a reduction of this sensor’s specificity. Based on the individual results of the BiDz sensors, the specificity, sensitivity, and accuracy of the BiDz-TB/NTM were 99.6%, 100%, and 0.99, respectively.

**TABLE 2:** BiDz sensor evaluation: Specificity, sensitivity, accuracy and LoD

Sensor	Specificity (%)^*a*^	Sensitivity (%)^*a*^	Accuracy^*a*^	Last detected concentration (pg/µL)^*b*^	LoD (genome copies)^*b*^
Mtb-BiDz	100	100	1.0	0.06	12
Mab/Mche-BiDz	100	100	1.0	≤0.03	≤5.23
Mav-BiDz	100	100	1.0	3.66	684
Mint/Mchi-BiDz	98	100	0.98	0.11	19.6
Mkan-BiDz	100	100	1.0	14.65	2,110
BiDz-TB/NTM	99.6	100	0.99	–	–

^*a*^ Sample *n*: 47 strains (41 mycobacterial strains and 6 strains of Gram-positive and Gram-negative bacteria). Specificity: rate of true negatives; sensitivity: rate of true positives; accuracy: fraction of the correctly identified samples.

^*b*^ Sample *n*: 20 serial twofold dilutions for each BiDz sensor.

All sensors detected their target species, with the LoD ranging from 5.23 to 2,110 genome copies (Table 2), which is compatible with typical bacterial loads in clinical samples. The Mab/Mche-BiDz showed the lowest LoD (≤5.23 genome copies), producing fluorescent signals at all dilutions evaluated. The Mtb-BiDz was able to produce strong fluorescent signals in up to 0.06 pg/µL of DNA, showing an LoD of 12 genome copies. The Mint/Mche-BiDz produced strong fluorescent signals in up to 0.11 pg/µL of DNA, showing an LoD of 19.6 genome copies. The Mav-BiDz produced strong fluorescent signals in up to 3.66 pg/µL of DNA, showing an LoD of 684 genome copies. Finally, the Mkan-BiDz showed the highest LoD (2,110 genome copies), requiring higher concentrations of DNA to produce positive fluorescent signals (14.65 pg/µL).

## DISCUSSION

The development of new diagnostic methods to differentiate *M. tuberculosis* and NTM infection rapidly and accurately is crucial for the introduction of effective therapeutic schemes, obtaining a favorable clinical outcome, and a robust public health surveillance (32, 38). The BiDz-TB/NTM proved capable of detecting and differentiating *M. tuberculosis* complex, MABC, *M. chelonae*, *M. avium*, *M. intracellulare*, *M. chimaera*, and *M. kansasii* (38).

The sequence of 16S rRNA has been widely used to differentiate bacterial species due to the presence of nine hypervariable regions (V1–V9), with a high nucleotide diversity, interspersed by conserved regions that are ideal targets for universal primer design. Although no hypervariable region can differentiate all bacterial species; the V2 hypervariable region has been considered the best differentiating region for mycobacterial species (43). It is a region of approximately 100 base pairs, covering 13 variable nucleotides among the species detected and identified by the method, ensuring their differentiation with high specificity and sensitivity (43, 44).

To achieve adequate sensitivity to detect mycobacteria in clinical samples, which present challenges such as low bacterial loads (e.g., sputum from juvenile patients or people living with HIV), nucleic acid-based POC assays often require amplification of target DNA. Hybridization-based detection technologies like BiDz sensors perform poorly on double-stranded DNA analytes generated by conventional symmetric PCR. As corroborated by our results with the R2 primer, even typical asymmetric PCR reactions using primers designed according to guidelines intended for symmetric PCR are very inefficient. To address this problem, we exploited the extensive work by Wangh *et al*. (36, 37) to develop a LATE-PCR method for optimal asymmetric amplification of the V2 hypervariable region of the mycobacterial 16S rRNA gene. Data presented herein demonstrate that simply increasing the length of the limiting primer dramatically enhances the generation of single-stranded amplicons detectable by BiDz sensors. In future studies, we will further validate the utility of the LATE-BiDz platform for detection of genetic mutations associated with antibiotic resistance in *M. tuberculosis*.

Conventionally, real-time PCR-based methods use short fluorescent probes (approximately 20–30 nucleotides) to maintain stability of the probe-target complex. However, this strategy can reduce the specificity of the diagnostic method. The BiDz-TB/NTM approach seeks to overcome this limitation by utilizing the BiDz sensors of approximately 40 nucleotides, divided into two subunits [Dz_a_ and Dz_b_ (BiDz)]. The splitting of the target-interrogated fragment of the probe into two and the need of both fragments to form near-perfect hybrids with the target for the signal guarantee a high diagnostic specificity (38, 45).

The specificity and sensitivity values of the BiDz sensors obtained were higher than those of other diagnostic methods that use DNA from mycobacterial cultures, such as line probe assay (LPA) platforms (46–48), with specificity of 99.6% and 94.1-100% and sensitivity of 100% and 100%, respectively. Furthermore, it takes 5–6 hours to obtain results by these methods, in contrast to the 1.5-hour sample-to-result time for BiDz assays. In fact, these methods have not been widely accepted due to the complexity of the protocols, which makes it restricted to reference laboratories (46, 48, 49). In addition, the specificity (99.6%), sensitivity (100%), and accuracy (0.99) values of the BiDz-TB/NTM reached the limits recommended by the WHO for new molecular diagnostic methods (35).

Despite the lack of differentiation between MABC and *M. chelonae* species by Mab/Mche-BiDz, these species are phylogenetically close. Until 2009, *M. abscessus* belonged to the *M. chelonae-abscessus* complex due to the high similarity with *M. chelonae* (17, 18), which differs by only four nucleotides in the 16S rRNA sequence. Furthermore, although these species are associated with skin, bone, and soft tissue infections (7), which require the same treatment, pulmonary infections caused by *M. chelonae* are rare (20).

The detection and non-differentiation of *M. intracellulare* and *M. chimaera* by Mint/Mchi-BiDz were predictable due to the variation of only one nucleotide in the final region of the 16S rRNA sequence and, therefore, outside the sensor complementarity location. Although the laboratory differentiation of these species is of epidemiological interest, clinical discrimination of *M. intracellulare* and *M. chimaera* is of lesser significance because the same therapeutic schemes are used for infectious diseases caused by both species (7, 50, 51). If differentiation of the two species is nevertheless required, it can be achieved by shifting the sensor’s site of interrogation to the fragment of 16S rRNA with nucleotide difference between the species. Indeed, discrimination of single nucleotide polymorphisms (SNPs) in genes associated with Mtb drug resistance by the BiDz sensors has been previously demonstrated (45).

Finally, it is noteworthy that this method was able to differentiate closely related non-target species. For example, despite 99.2% similarity in the 16S rRNA sequence of *M. szulgai* and MAC species (52), it was correctly discriminated by the Mav-BiDz and Mint/Mchi-BiDz sensors. Genetically similar slow-growing mycobacteria *M. tuberculosis* and *M. kansasii* (24, 25, 53, 54) were also correctly differentiated when the Mtb-BiDz and Mkan-BiDz sensors were used.

Our results demonstrate that the BiDz-TB/NTM, using flexible, low-cost, and easy to synthesize sensors, is promising for the development of a POC diagnostic platform for accurate screening of infections caused by the main mycobacteria of clinical interest. However, to meet the demands of a POC platform, BiDz TB/NTM, currently performed in two steps—PCR amplification and BiDz assay—will need to be improved and evaluated with a greater number of samples, in addition to being implemented on a portable device, an important part of a POC test (55, 56). Furthermore, to develop the full diagnostic potential of the proposed approach, assays demonstrating its feasibility for clinical samples are required and are scheduled as the next phase of development of the BiDz TB/NTM method.

Current limitations of the assay include a two-step format, which requires manual addition of the amplicons to the components of the BiDz sensors. This may increase the probability of samples’ cross-contamination and adds to the hands-on time. An ideal format would be either a one-pot amplification and detection step or use of a microfluidics device to automatically deliver the amplicon to the sensors. Another limitation is the need for a thermocycler to perform the nucleic acid amplification step. This limitation can be offset by access to the portable three-dimensional-printed thermal cycler that is under development. Finally, the limitation of the technology in its current format is the need to isolate bacterial DNA from clinical samples. This limitation, however, is shared by most of the nucleic acid amplification tests and can be mitigated by integration of the sample processing step into the microfluidics device, which is envisioned to be the final format of the assay for its use in clinical practice.

## SUPPLEMENTAL MATERIAL

Supplemental material spectrum.03506-23-s0001.docx Table S1 (clinical strains included in study); Table S2 (BiDz sensor oligos and substrate); Figure S1 (16S rRNA sequence alignments).

Fig. S2 spectrum.03506-23-s0002.xlsx Supplemental graphs showing BiDZ of all clinical isolates individually. Full data set corresponding to Fig. 3.

ASM does not own the copyrights to Supplemental Material that may be linked to, or accessed through, an article. The authors have granted ASM a non-exclusive, world-wide license to publish the Supplemental Material files. Please contact the corresponding author directly for reuse.
