# Supplementary material for: Detection of Mtb and NTM: preclinical validation of a new asymmetric PCR-binary deoxyribozyme sensor assay
Source: Microbiol Spectr. 2024 Apr 23;12(6):e03506-23. doi: 10.1128/spectrum.03506-23 (PMC11237447; doi:10.1128/spectrum.03506-23)
Supplement: Supplemental material — Table S1 (clinical strains included in study); Table S2 (BiDz sensor oligos and substrate); Figure S1 (16S rRNA sequence alignments). [file spectrum.03506-23-s0001.docx]

**SUPPLEMENTARY MATERIAL:**

**Table 1S:** Clinical strains of *Mycobacterium* sp. Identification

| **Species** | **Number of strains** | **Identification** |
| --- | --- | --- |
| *M. tuberculosis* | 1  5 | H37Rv strain*  MIRU-VNTR 24 *loci;* IS*6110* and WGS |
| *M. abscessus* subsp. *abscessus* | 5 | *rpoB* and *hsp65* genes partial sequencing |
| *M. abscessus* subsp. *bolletii* | 5 | *rpoB* and *hsp65* genes partial sequencing |
| *M. abscessus* subsp. *massiliense* | 5 | *rpoB* and *hsp65* genes partial sequencing |
| *M. avium* | 1  5 | ATCC 03057HC*  *rpoB* and *hsp65* genes partial sequencing |
| *M. intracellulare* | 4 | *rpoB* and *hsp65* genes partial sequencing |
| *M. kansasii* | 1  4 | ATCC 12478*  *rpoB* and *hsp65* genes partial sequencing |
| *M. fortuitum* | 1 | *rpoB* and *hsp65* genes partial sequencing |
| *M. marinum* | 1 | ATCC 927* |
| *M. chelonae* | 1 | ATCC 946* |
| *M. chimaera* | 1 | *rpoB* and *hsp65* genes partial sequencing |
| *M. szulgai* | 1 | ATCC 10831* |

*No molecular identification methods were performed as they were reference strains. **MIRU-VNTR**: Mycobacterial Interspersed Repetitive Unit-Variable Number Tandem Repeat; **WGS**: Whole Genome Sequencing.

**Table 2S:** Sequences for the sensor strands and fluorogenic substrate used in the study.

| **BiDz sensors*** | **Sequence** |
| --- | --- |
| Dz_a__16S_Mtb Dz_b__16S_Mtb | 5’ - TGCCCAGGGA*GGCTAGCT*GGTCCTATCCGGTATTAGACCC - 3’  5’ - CACAAGACATGCATCCCGT*ACAACGA*GAGGAAACCTT - 3’ |
| Dz_a__16S_Mab  Dz_b__16S_Mab | 5’ - TGCCCAGGGA*GGCTAGCT*AGTGTGTGGTCCTATCCGGT - 3’  5’ - AAAAGCTTTGCACCACTCACCATGA*ACAACGA*GAGGAAACCTT - 3’ |
| Dz_a__16S_Mav  Dz_b__16S_Mav | 5’ - TGCCCAGGGA*GGCTAGCT*GTCTTGAGGTCCTATCCGGTAT - 3’  5’ - TCCACCAGAAGACATGC*ACAACGA*GAGGAAACCTT - 3’ |
| Dz_a__16S_Mint  Dz_b__16S_Mint | 5’ - TGCCCAGGGA*GGCTAGCT*ATGCGCCTAAAGGTCCTAT - 3’  5’ - AAAAGCTTTCCACCTAAAGAC*ACAACGA*GAGGAAACCTT - 3’ |
| Dz_a__16S_Mkan  Dz_b__16S_Mkan | 5’ - TGCCCAGGGA*GGCTAGCT*GGTCCTATCCGGTATTAGACCC - 3’  5’ CAAGGCATGCGCCAAGT*ACAACGA*GAGGAAACCTT – 3’ |
| **Fluorescent substrate** | **Sequence** |
| MzF-FAM | 5’ - AAGGT-(dT-FAM)-TCCTCguCCCTGGGCA-(BHQ) - 3’ |

*Mtb: *M. tuberculosis* complex; Mab: *M. abscessus* complex; Mav: *M. avium*; Mint: *M. intracellulare*; Mkan: *M. kansasii*; FAM – fluorescein, BHQ – Black Hole Quencher 1; nucleotides corresponding to the catalytic core of 10-23 deoxyribozyme are in italic, nucleotides of the substrate-binding fragments of the sensor strands and the corresponding substrate fragments are color-coded; ribonucleotides are in lowercase.


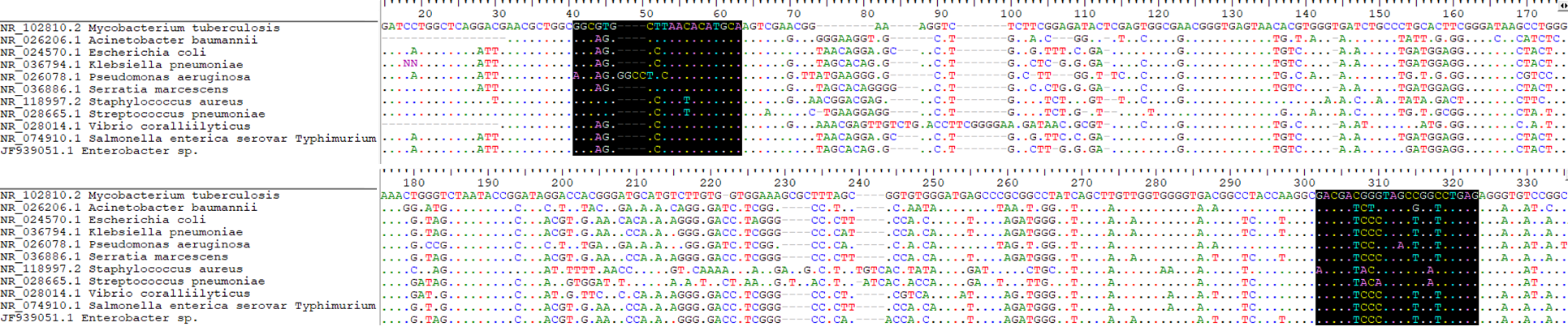


**Fig. 1S:** Alignment of 16S ribosomal RNA sequences from different bacterial species against *Mycobacterium tuberculosis H37Rv*. Marked in black are the locations of the forward and reverse primers on the 16S ribosomal RNA sequences (5'-3'). The dots indicate nucleotides conserved with *M. tuberculosis H37Rv*, while the dashes indicate insertions in one or more sequences that result in gaps in the alignment. This highlights the uniqueness of the primer sequences with multiple mismatches compared to other species as well as the many *Mtb* specific polymorphisms within this hypervariable region of 16S rRNA. The target sequence of Dz_a_ sensor is indicated by the green bar (nt 181-202), and Dz_b_ sensor is designed to hybridize to sequence indicated by the orange bar (nt 203-221) in the alignment.
